# Protection of bioreactor culture from virus contamination by use of a virus barrier filter

**DOI:** 10.1186/1753-6561-9-S9-P22

**Published:** 2015-12-14

**Authors:** Kimberly Mann, Jon Royce, Christina Carbrello, Robert Smith, Rong-Rong Zhu, Yuanchun Zeng, Anne Leahy, Mary Priest, Ven Raman, Jayant S Nick Harrington, Scott Bliss, William Bryant, Nhung Nguyen, Soleil Le, Danielle DeCesaro, Jeremy Perreault, Philip Goddard, Joe Orlando, Kevin Rautio

**Affiliations:** 1 EMD Millipore Corporation, Bedford, MA, 01730, USA

## Background

Protection of bioreactors from viruses and mycoplasma remains a challenge. While the risk of these contaminations is known, and several recent high profile cases have raised awareness of this risk, many bioreactors remain unprotected. Currently available technologies, such as high-temperature short-time treatment and ultraviolet light treatment, can be difficult to implement due to footprint, efficacy, and media compatibility issues. Size exclusion nanofiltration may be a more ideal technology for virus and mycoplasma protection, potentially offering advantages in robustness, scalability, small footprint, and media compatibility.

However, current virus removal filters, designed for monoclonal antibody purification, are generally poorly suited for cell culture media processes due to the large membrane surface areas needed to achieve adequate flow and capacity in a reasonable timeframe. Here we evaluate a novel virus barrier filter specifically developed for cell culture media applications.

## Materials and Methods

The Viresolve®Barrier filter was evaluated for retention of virus, bacteria and mycoplasma and for effects on cell culture growth and product quality. Flow rate and capacity were benchmarked against existing commercially available virus membranes in order to compare both the performance and economics for the filtration of cell culture media and feeds.

Retention of microorganisms was performed at constant pressure with both media and buffer utilizing seven microorganisms (viruses, bacteria and mycoplasma). Retention testing was performed in lab scale devices with typical membrane samples expected to give representative performance.

To demonstrate cell culture performance, Cellvento® CHO-200 media and the corresponding feeds were processed through a Viresolve® Barrier filter. 1H-NMR at 500 MHz, inductively coupled plasma/optical emission spectrometry (ICP/OES) and reverse-phase LC-MSMS were used to assess any effects of filtration on media components. Fed batch studies were performed in shake flask cultures using recombinant mAb producing CHO cells. Cell culture performance and protein quality were evaluated and compared to a 0.2 µm Millipore Express® filtered control.

## Results

Preliminary testing has shown high retention for small viruses as well as complete removal (up to detection limit) of large viruses, bacteria and mycoplasma (Table 1). Both standard and difficult-to-retain bacteria and mycoplasma were tested. As the filter is gamma stable and steam in place (SIP) compatible it could be used in place of the standard 0.2 µm filter.

**Table 1:** Microorganism retention testing usingViresolve® Barrier filter.

Type	Organism	Description	TypicalLog Reduction Value
Viruses	Minute Virus of Mice (MVM)	Model parvovirus, reported contaminant of manufacturing operations, target organism	4.8
	Phi-X174	Model bacteriophage of similar size to parvovirus, used as surrogate	6.0
	XentropicMurineLeukemia Virus (xMuLV)	Model retrovirus	>5.3
Bacteria	*Brevundimonasdiminuta*	Standard model bacteria for ASTM sterilizing grade designation	>8.0
	*Leptonemaillini*	Model spirochete bacteria, difficult to retain on sterilizing grade filters	>8.0
Mycoplasma	*Acholeplasmalaidlawii*	Standard model mycoplasma, pleomorphic, retained by 0.1 µm filters	>8.0
	*Mycoplasma orale*	Model mycoplasma, small and pleomorphic, difficult to retain on 0.1 µm filters	>8.0

Virus filtration is often perceived as unsuitable for cell culture media due to its high cost and large footprint. Over a six hour filtration process, Viresolve®Barrier filter had a volumetric throughput approximately 3 to 30 times higher than other commercially available virus filters.

**Figure 1:**
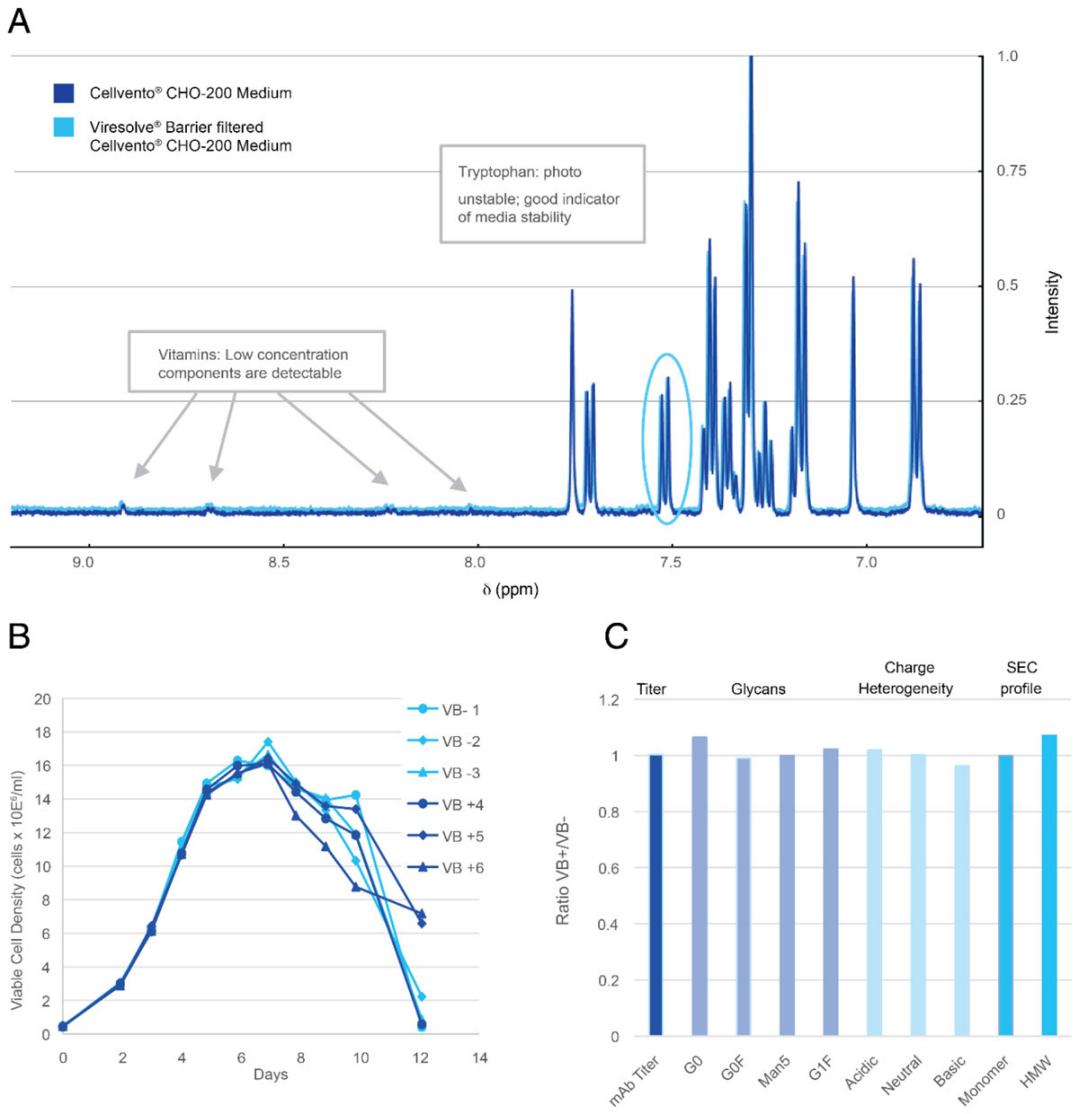
**(a) NMR fingerprinting of media pre-filtration (dark blue) or post Viresolve®Barrier filtration (light blue) showed no change in media composition**. (b): Cell growth of fed-batch cultures using Viresolve®Barrier filtered media and feeds (VB+) or 0.22µM filtered media and feeds (VB-). (c): Product quality of the mAb is unchanged by virus filtration of the media and feeds. All attributes displayed as a ratio of VB+/VB-.

It is crucial that a virus barrier filter does not remove any critical media components or impact cell culture performance. A multidimensional analysis of the performance of filtered media in recombinant mAb-producing cell cultures revealed no impact. Media and feed components were unaffected by filtration through the Viresolve®Barrier filter as evaluated by 1H-NMR, ICP/OES and LC-MSMS. Cultures showed no differences in cell growth or titer relative to the sterile filtered control. Furthermore secreted antibody showed no differences in aggregation profiles, charge variants or glycosylation patterns (Figure 1).

## Conclusions

The risk of virus contamination in the bioreactor remains a concern for biotherapeutic manufacturers as no single technology provides sufficient, economical protection while minimizing the impact to cell culture. This study evaluated a virus barrier filter that provides an efficient and easy way to protect a bioreactor from adventitious virus.

Study results demonstrated high retention for small viruses and detection limit retention of large viruses, mycoplasma and bacteria, while requiring less than half the area of commercial virus filters. Since the filter is stable to gamma and SIP sterilization, it could be used in place of a sterile filter. Favorable cell culture performance and extensive analytical analysis indicated little change in the media composition after virus barrier filtration. The protein quality attributes were equivalent to those from a control process using standard 0.2 µm filtration.

Results from this study suggest that filtration with a Viresolve®Barrierfilter can provide optimal filtration performance, high retention, and minimal cell culture impact as well as providing a viable option to improve the overall virus safety strategy for chemically defined cell culture media.
